# Supplementary material for: Occurrence of Calcium Oscillations in Human Spermatozoa Is Based on Spatial Signaling Enzymes Distribution
Source: Int J Mol Sci. 2021 Jul 27;22(15):8018. doi: 10.3390/ijms22158018 (PMC8347727; doi:10.3390/ijms22158018)
Supplement: Supplementary file 1 [file ijms-22-08018-s001.zip › ijms-1279186-supplementary.pdf]

Supporting information

Occurrence of calcium oscillations in human spermatozoa is based on spatial signaling enzymes distribution

Julia Korobkin[1,2], Fedor A. Balabin[2], Sergey A. Yakovenko[1,3], Ekaterina Simonenko[1], and Anastasia N. Sveshnikova[1,2,4*]

[1] Faculty of Physics, Lomonosov Moscow State University, 1/2 Leninskie gory, Moscow, 119991, Russia

[2] Center for Theoretical Problems of Physico-Chemical Pharmacology, Russian Academy of Sciences, 30 Srednyaya Kalitnikovskaya str., Moscow, 109029, Russia

[3] AltraVita IVF and gynecology clinic, 4A, Nagornaya st., Moscow, 117186, Russia

[4] Department of Normal Physiology, Sechenov First Moscow State Medical University, 8/2 Trubetskaya St., Moscow, 119991, Russia

*To whom correspondence should be addressed: and Anastasia N. Sveshnikova, a.sveshnikova@physics.msu.ru, https://orcid.org/ 0000-0003-4720-7319.

Keywords: CatSper, sperm cell, calcium signaling, progesterone, computational modeling, reaction-diffusion system

Abbreviations 2

S1. Calcium concentration measurement in a single cell 3

Figure S1. Measurement of calcium concentration in a single sperm cell 3

S2. Geometrical region details 4

Table S1. Three-dimensional model geometry 4

Figure S2. Three-dimensional model geometry. 4

S3. Three-dimensional model design. CatSper module 5

Table S2. Three-dimensional model design. CatSper module constants 5

S4. CatSper module simulation results 6

Figure S3. Simulation of CatSper channel flux dependence on progesterone concentration 7

Figure S4. Simulation of CatSper channel open state probability dependence on time upon activation with 5 µM progesterone 7

S5. Three-dimensional model design. Calcium module 7

Table S3. Calcium signaling module parameters 9

S6. Homogeneous model design 11

Figure. S5. Homogeneous model design 11

S7. Quasi-heterogeneous model 11

Figure S6. Scheme of the quasi-heterogeneous model. 12

Figure S7. Quasi-heterogeneous model computational experiment on progesterone activation of sperm cells. 12

S8. Protein number estimation 12

S9. Calculating the change in channel flux upon addition of nominally calcium-free buffer 13

Figure S8. Proof of the validity of PLCd inclusion in the model 14
S10. Discussion on participation of PLCδ in progesterone activation of human cells 14
S11. Parameter scan for the homogeneous model 14
Figure S9. Dependence of calcium oscillation maximal concentration on the oscillation frequency for different model parameter value....15
Figure S10. Vitality check for the spermatozoa adherent to poly-L-lysine 15
S12. Adhesion of different populations of sperm cells to poly-L-lysine 15
S13. Supporting references....16

Abbreviations

2-AG — 2-arachidonoylglycerol

AA — arachidonic acid

ABHD2 — alpha-beta-hydrolase 2

CaM — calmodulin

CaRet — calreticulin

IP_3 — inositol-1,4,5-trisphosphate

IP5pp — Inositol-polyphosphate 5-phosphatase

MP — midpiece

PIP_2 — phosphatidylinositol 4,5-bisphosphate

PLCδ — phospholipase Cδ

PMCA — plasma membrane calcium ATPase

PP — principal piece

SPCA — signaling pathway calcium ATPase

S1. Calcium concentration measurement in a single cell

Measurement of cytosolic calcium concentration was conducted in flow chambers by low-angle fluorescent microscopy. First, a flow chamber [1] was assembled using a Poly-L-lysine coated cover glass (Fig. S1). Afterwards, AllGrad Wash® buffer was pumped into the flow chamber with a syringe pump, and the flow chamber was mounted on the microscope. Then a suspension of Fura-Red loaded spermatozoa in AllGrad Wash® buffer was perfused through the chamber with a syringe pump and left for 20 minutes to let the cells attach to poly-L-lysine. Then unattached cells were removed by AllGrad Wash® buffer. Then the baseline signal was taken. Afterwards, the buffer was changed to 5 μM progesterone solution in AllGrad Wash® to induce cells activation.

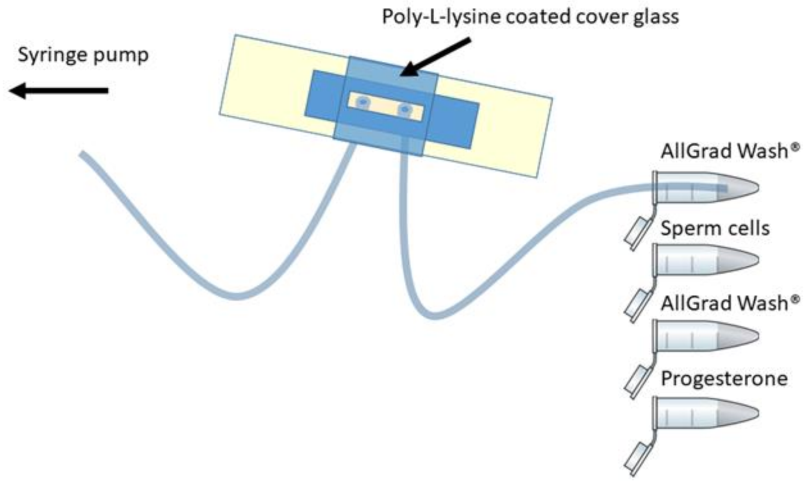

Figure S1. Measurement of calcium concentration in a single sperm cell. Calcium concentration in single cells was measured using the flow chambers with a poly-L-lysine coated glass. First, the flow chamber was filled with AllGrad Wash® and mounted on the microscope. The tubes are lined in the same order as the solutions were perfused through the chamber.

S2. Geometrical region details

The three-dimensional model was constructed using VCell software (http://www.vcell.org/). Corresponding Virtual Cell model, SpermCalcium, is available in the public domain at http://www.vcell.org/ under the shared username "Juliajessica." The geometric region details are given in Table S1.

The geometry of the three-dimensional model consists of extracellular space (300 μm^3), sperm cytosol (18.4 μm^3) divided into sperm tail (0.4 μm in diameter [2], 46 μm long [3]), sperm midpiece MP (0.8 μm in diameter [2], 7 μm long [4]) and sperm head (4.5 x 3 x 1.5 μm [4]), and sperm calcium store RNE [5] (0.11 μm^3) located between sperm head and sperm MP (Fig. S2). The extracellular region was modeled as a 5x5x55 box with species concentration (progesterone) set to be constant.

Table S1. Three-dimensional model geometry

Domain name	Domain eq.	Domain volume, μm^3	Domain membrane, μm^2	Reference
Cytosol	((((x >= - 12.0) && (x <= 28.0) && ((((y - 5.0) ^ 2.0) + ((z - 5.0) ^ 2.0)) < (0.25 ^ 2.0))) + ((((x - 34.8) ^ 2.0) + (((1.4 * y) - (1.4 * 5.0)) ^ 2.0) + (((2.0 * z) - 10.0) ^ 2.0)) < 3.0)) + ((x >= 28.0) && (x <= 34.0) && ((((y - 5.0) ^ 2.0) + ((z - 5.0) ^ 2.0)) < (0.4 ^ 2.0))))	18.4	84.4	[6]
Redundant nuclear envelope (RNE)	((((x - 33.6) ^ 2.0) + ((y - 5.0) ^ 2.0) + ((z - 5.0) ^ 2.0)) < (0.3 ^ 2.0))	0.11	1.8	[5]

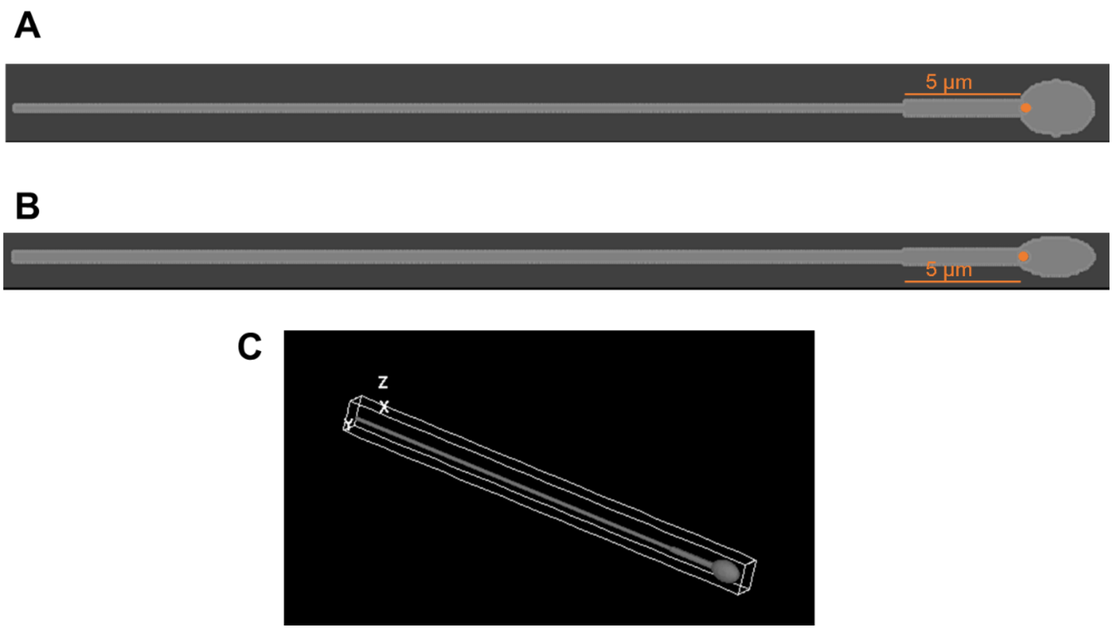

Figure S2. Three-dimensional model geometry. (A) The central 2D slice of the cell (YZ projection). The model geometry consists of three compartments: RNE (red), cytosol (light gray), and extracellular space (dark gray). (B) The central 2D slice of the cell (XZ projection). (C) The three-dimensional sperm cell geometry

S3. Three-dimensional model design. CatSper module

The first module of the 3D model focuses on the CatSper channel opening dynamics upon treatment of spermatozoa with progesterone. All of the reactions occur on the plasma membrane. In resting state, the CatSper was considered to be inhibited by plasma membrane lipid 2-arachidonoylglycerol (2-AG). Progesterone activates alpha-beta-hydrolase 2 (ABHD2), an enzyme that cleaves 2-AG into arachidonic acid (AA) and glycerol and thus activates CatSper. The sperm plasma membrane constantly produces 2-AG in order to keep CatSper closed [7].
Concentration dynamics and diffusion of CatSper channel protein, ABHD2, 2-AG, and AA were regarded. Localization and diffusion of ABHD2 and CatSper are assumed to be restricted to sperm principal piece (PP) [3]. The annulus limits protein diffusion in mature spermatozoa. It is a cytoskeletal structure located between the MP and PP [4]. AA and 2-AG molecules were assumed to be evenly distributed in the plasma membrane. ABHD2 gets activated upon binding to a progesterone molecule from the extracellular space. The following equations (1)-(7) were implemented in VCell to describe CatSper channel activation:

$$\frac{\partial[ABHD^*]}{\partial t} = k_{+ABHDProg}[AB\mathrm{HD}][\mathrm{Progesterone}] - \frac{k_{+ABHDProg}}{K_{\mathrm{mABHDProg}}}[ABHD^*] + D_{Pr}\Delta[ABHD^*] \quad (1)$$

$$\frac{\partial[CatSper]}{\partial t} = -k_{+CatSper}[CatSper][\mathrm{AG}] + \frac{k_{+CatSper}}{K_{\mathrm{mAGCsp}}}[\mathrm{CatSperAG}] + D_{Pr}\Delta[CatSper] \quad (2)$$

$$\frac{\partial[ABHD]}{\partial t} = -k_{+ABHDProg}[AB\mathrm{HD}][\mathrm{Progesterone}] + \frac{k_{+ABHDProg}}{K_{\mathrm{mABHDProg}}}[ABHD^*] + D_{Pr}\Delta[ABHD] \quad (3)$$

$$\frac{\partial[CatSperAG]}{\partial t} = k_{+CatSper}[CatSper][\mathrm{AG}] - \frac{k_{+CatSper}}{K_{\mathrm{mAGCsp}}}[\mathrm{CatSperAG}] + D_{Pr}\Delta[CatSperAG] \quad (4)$$

$$\frac{\partial[AG]}{\partial t} = -\frac{k_{catABHD}[AG][ABHD^*]}{K_{\mathrm{mABHD-AG}} + [AG]} + [AA]\frac{1}{t_{aa}} -$$

$$-k_{+CatSper}[CatSper][\mathrm{AG}] + \frac{k_{+CatSper}}{K_{\mathrm{mAGCsp}}}[\mathrm{CatSperAG}] + D_{AG}\Delta[AG] \quad (5)$$

$$\frac{\partial[AA]}{\partial t} = \frac{k_{catABHD}[AG][ABHD^*]}{K_{\mathrm{mABHD-AG}} + [AG]} - \frac{1}{t_{aa}}[AA] + D_{AA}\Delta[AA] \quad (6)$$

$$D_{Pr} = \begin{cases} D_{Prot}, & \vec{x} \in PP \\ 0, & \vec{x} \notin PP \end{cases}, \quad (7)$$

where $[ABHD]$ denotes the non-activated ABHD2 surface concentration, $[ABHD^*]$ is the activated ABHD2 surface concentration, $[AG]$ denotes 2-AG surface concentration, $[AA]$ is AA surface concentration, $[CatSper]$ is open CatSper channel surface concentration, $[CatSperAG]$ is closed CatSper channel surface concentration. Equation (7) defines the diffusion coefficients of all listed proteins in the plasma membrane. Parameters for the equations (1)-(7) are given in Table S2.

Table S2. Three-dimensional model design. CatSper module constants

	Value	Reference	Comment
$kcat_{ABHD}$	10	This work	Turnover speed for 2-AG cleavage by ABHD

$K_{mABHD\text{-}AG}$	12 µM	[8]	ABHD2 affinity for 2-AG
$N_{CatSper}$	100	S8	Initial CatSper molecule number
K_{mAGCsp}	0.67	[7]	2AG affinity for CatSper
$k_{+ABHDProg}$	3	This work	k_+ for binding progesterone to ABHD2
$K_{mABHDProg}$	16	[7]	ABHD2 affinity for the progesterone
$k_{+CatSper}$	10	This work	Binding speed of 2AG to CatSper
t_{AA}	1.67 min	[7]	Arachidonic acid generation rate.
$ABHD_0$	100	S8	Initial ABHD2 molecule number
$2AG_0$	0.7 µM	[7]	Initial 2AG concentration
D_{Prot}	2 µm^2/s	[9]	The diffusion coefficient for the proteins in the membrane
D_{AA}	1 µm^2/s	[10]	AA diffusion coefficient
D_{AG}	1 µm^2/s	[10]	2-AG diffusion coefficient

S4. CatSper module simulation results

The model prediction for the dependence of flux through CatSper on progesterone concentration is given in Figure S3. Half-maximal activation was reached at 6 nM progesterone, which is consistent with experimental data [11]. The CatSper module also described the dynamics of the channel activation by saturating progesterone concentrations (Fig. S4). It was approximated with a step function:

$$J_{CatSper} = \begin{cases} V_{max}, t > t_{prog} \\ 0, t > t_{prog} \end{cases},$$

where V_{max} denotes maximum CatSper conductivity, and t_{prog} denotes progesterone application time.

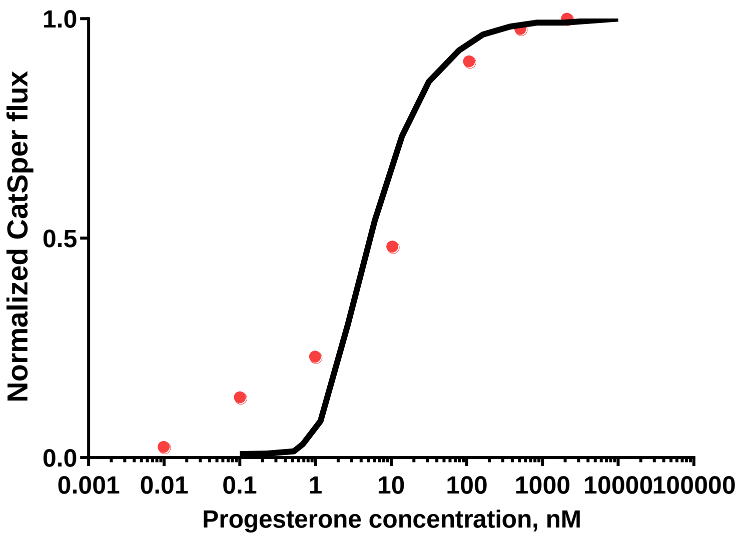

Figure S3. Simulation of CatSper channel flux dependence on progesterone concentration. Dependence of open CatSper channels normalized flux on progesterone concentration. The line represents simulation data. Red dots represent data from [7].

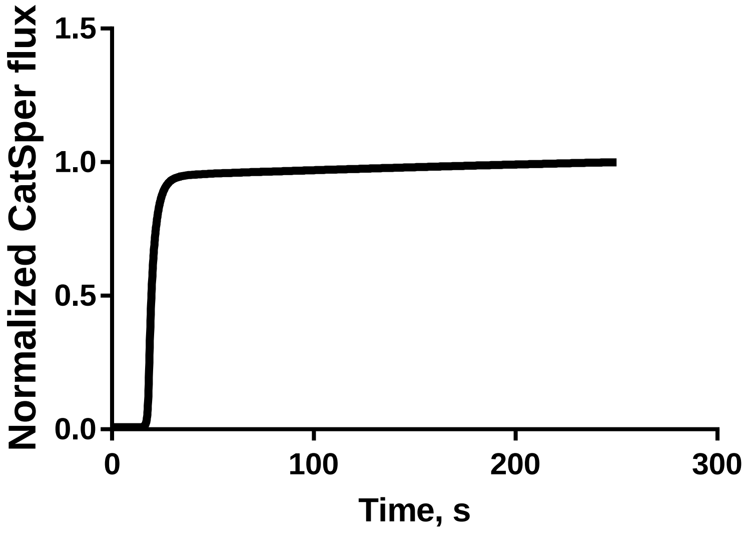

Figure S4. Simulation of CatSper channel open state probability dependence on time upon activation with 5 μM progesterone. Model CatSper response to activation with progesterone. Model progesterone concentration is set equal to 5 μM at $t_{prog} = 25$ s. Channel flux change upon progesterone application can be approximated with a step function.

S5. Three-dimensional model design. Calcium module

Calcium ions enter cytosol through open CatSper channels and then diffuse into spermatozoon MP, where a specific isoform of phospholipase C, PLCδ, is located. This enzyme activity is upregulated by calcium concentration rise; PLCδ catalyzes inositol-1,4,5-trisphosphate (IP_3) production from a membrane phospholipid phosphatidylinositol 4,5-bisphosphate (PIP_2). IP_3 activates inositol trisphosphate receptors (IP3R) located on the spermatozoon calcium store redundant nuclear envelope (RNE), and thus induces calcium release into cell cytoplasm through those receptors. Plasma membrane calcium ATPase (PMCA) present in the sperm cilia membrane extrudes calcium into extracellular media. Signaling pathway

calcium ATPase (SPCA) pumps calcium into calcium store RNE. The following equations (8),(9) describe calcium dynamics in cytosol and RNE:

$$\frac{\partial[Ca^{2+}_{Cyt}]}{\partial t} = J_{RNE,leak} - J_{SPCA} - J_{PMCA} + J_{IP3R} + J_{leak} + J_{catsper} + J_{BufCyt} + D_{ca}\Delta[Ca^{2+}_{Cyt}] \quad (8)$$

$$\frac{\partial[Ca^{2+}_{ER}]}{\partial t} = \beta J_{SPCA} - \beta J_{RNE,leak} - \beta J_{IP3R} + J_{BufER} + D_{caER}\Delta[Ca^{2+}_{ER}], \quad (9)$$

where $[Ca^{2+}_{Cyt}]$ denotes cytosolic calcium concentration, $[Ca^{2+}_{ER}]$ is the RNE calcium concentration. J_{SPCA} is the calcium flux mediated by SPCA. SPCA is located on the RNE membrane [12]. J_{PMCA} is calcium flux mediated by PMCA. PMCA activity is assumed to be non-zero only in sperm PP [13]. J_{IP3R} is calcium flux through IP3R located on the RNE membrane, J_{leak}, and $J_{RNE,leak}$ are calcium leaks. The RNE has spherical geometry and calcium ($J_{RNE,leak}$,J_{SPCA},J_{IP3R}) is released uniformly on the boundary of the sphere. Calcium leak from the outer medium is restricted to PP [14]. J_{BufER} and $J_{BufCytosol}$ stand for calcium buffering in RNE and cytosol. The equations for the Calcium module (8)-(9) fluxes are the following:

$$J_{SPCA} = \begin{cases} \frac{V_{SPCA}[Ca^{2+}_{Cyt}]^{n_{hillspca}}}{K_{SPCA}{}^{n_{hillspca}} + [Ca^{2+}_{Cyt}]^{n_{hillspca}}}, & \vec{x} \in RNEmembr \\ 0, & x \notin RNEmembr \end{cases}, V_{SPCA} = \frac{k_{catSPCA}N_{SPCA}}{V_{Cyt}N_A} \quad (10)$$

$$J_{PMCA} = \begin{cases} \frac{V_{PMCA}[Ca^{2+}_{Cyt}]^{n_{hill}}}{K_{PMCA}{}^{n_{hill}} + [Ca^{2+}_{Cyt}]^{n_{hill}}}, & \vec{x} \in PP \\ 0, & \vec{x} \notin PP \end{cases} \quad (11)$$

$$J_{RNE,leak} = \begin{cases} k_{leak}([Ca^{2+}_{ER}] - [Ca^{2+}_{Cyt}]), & \vec{x} \in RNE \\ 0, & \vec{x} \notin RNE \end{cases} \quad (12)$$

$$J_{leak} = \begin{cases} k_{leakPP}, & \vec{x} \in PP \\ 0, & \vec{x} \notin PP \end{cases} \quad (13)$$

$$J_{BufER} = -[CaRetSlow][Ca^{2+}_{ER}]k^{+}_{retS} + [CaRetSlowCa]k^{-}_{retS} -$$

$$-[CaRetFast][Ca^{2+}_{ER}]k^{+}_{retF} + [CaRetFast]k^{-}_{retF} \quad (14)$$

$$J_{BufCyt} = -[CaMSlow][Ca^{2+}_{Cyt}]k^{+}_{MS} + [CaMSlowCa]k^{-}_{MS} - [CaMFast][Ca^{2+}_{Cyt}]k^{+}_{MF} + [CaMFast]k^{-}_{MF} \quad (15)$$

$$J_{CatSper} = \begin{cases} P_{CatSper}V_{CatSper}, & \vec{x} \in PP \\ 0, & \vec{x} \notin PP \end{cases}, \quad (16)$$

where the calcium buffering phenomenon is taken into account (eq. (14)). $\vec{x}$ is the coordinates of the current cell of the spatial mesh. Calcium buffering proteins such as calmodulin (CaM) located in cytosol and calreticulin (CaRet) located in the endoplasmic reticulum can bind calcium reversibly. Two noncooperative sites are included for each mobile buffer: sites C and P for CalRet and C and N for CaM [15]. The following equations describe concentrations of the calcium buffering proteins.

$$\frac{\partial[CaRetSlow]}{\partial t} == -[CaRetSlow][Ca^{2+}_{ER}]k_{+\mathrm{CaRetSlow}} + [CaRetSlowCa]\frac{k_{+\mathrm{CaRetSlow}}}{K_{\mathrm{dCaRetSlow}}} + D_{caER}\Delta[CaRetSlow] \quad (17)$$

$$\frac{\partial[CaRetFast]}{\partial t} == -[CaRetFast][Ca^{2+}_{ER}]k_{+\mathrm{CaRetFast}} + [CaRetFastCa]\frac{k_{+\mathrm{CaRetFast}}}{K_{\mathrm{dCaRetFast}}} + D_{caER}\Delta[CaRetFast] \quad (18)$$

$$\frac{\partial[CaMFast]}{\partial t} == -[CaMFast][Ca^{2+}_{Cyt}]k_{+CaMFast} + [CaMFastCa]k_{-CaMFast} + D_{ca}\Delta[CaMFast] \quad (19)$$

$$\frac{\partial[CaMSlow]}{\partial t} = = -[CaMSlow]\left[Ca^{2+}_{Cyt}\right]k_{+\mathrm{CaMSlow}} + [CaMSlowCa]\frac{k_{+\mathrm{CaMSlow}}}{K_{\mathrm{dCaMSlow}}} + D_{ca}\Delta[CaMSlow], \quad (20)$$

where $[CaMSlow]$ denotes calcium-free cytosolic CaM binding site N concentration, $[CaMFast]$ is calcium-free CaM binding site C concentration, $[CaRetSlow]$ is calcium-free CaRet binding site C concentration, $[CaRetFast]$ is the denotation for calcium-free CaRet binding site P concentration. $[CaMSlowCa]$ is calcium-bound cytosolic CaM binding site N concentration, $[CaMFastCa]$ denotes calcium-bound CaM binding site C concentration, $[CaRetSlowCa]$ denotes calcium-bound CaRet binding site C concentration, $[CaRetFast]$ is for calcium-bound CaRet binding site P concentration.

The flux through the IP3R was determined in accordance with the work of De Young and Keizer [16]

$$J_{IP3R} = \frac{\mathrm{k_{IP3R}N_{IP3R}}[Ca^{2+}_{ER}]P_0}{V_{cyt}}, \quad P_0 = P_{4o} + P_{3o} = w_{110}^4 + 4w_{110}^3(1-w_{110}) \quad (21)$$

$$w_{110} = \frac{[IP3]}{K_1}\frac{[Ca^{2+}_{cyt}]}{K_5}\frac{1}{Z},$$

$$Z = 1 + \frac{[Ca^{2+}_{RNE}]}{K_4} + \frac{[Ca^{2+}_{RNE}]}{K_5} + \frac{[Ca^{2+}_{RNE}]}{K_4}\frac{[IP3]}{K_5} + \frac{[IP3]}{K_1} + \frac{[IP3]}{K_1}\frac{[Ca^{2+}_{RNE}]}{K_2} + \frac{[IP3]}{K_1}\frac{[Ca^{2+}_{RNE}]}{K_5} + \frac{[IP3]}{K_1}\frac{[Ca^{2+}_{RNE}]}{K_2}\frac{[Ca^{2+}_{RNE}]}{K_5}, \quad (22)$$

where P_0 denotes IP3R open state probability, w_{110} is single IP3R subunit open-state probability (De Young and Keizer IP3R model is used, see [16] for details) Z is a normalization factor.

Then, the equation for IP3 and the activity of PLC will be:

$$\frac{\partial[IP3]}{\partial t} = \begin{cases} J_{PLC} - J_{IP3K} + D_{IP3}\Delta[IP3], & \vec{x} \in Midpiece, Head, \\ D_{IP3}\Delta[IP3] - J_{IP3K}, & \vec{x} \notin Midpiece, Head \end{cases} \quad (23)$$

$$J_{IP3K} = \frac{V_{IP3K}[\mathrm{IP3}]}{K_{IP3K} + [\mathrm{IP3}]} \quad (24)$$

$$J_{PLC} = \frac{V_{PLC}[Ca^{2+}_{Cyt}]}{K_{PLC} + [Ca^{2+}_{Cyt}]}, \quad (25)$$

$$V_{PLC} = \frac{k_{PLC}N_{PLC}}{V_{Cyt}N_A}, \quad (26)$$

where $[IP3]$ denotes IP_3 concentration. J_{IP3K} is the rate of IP_3 degradation by Inositol-polyphosphate 5-phosphatase (IP5pp). J_{PLC} is the rate of IP_3 generation by PLCδ, which activity is upregulated by calcium ion concentration rise. IP_3 generation is assumed to happen only in the sperm MP and head [14], [17]. The parameters of equations (8)-(26) are given in Table S3.

Table S3. Calcium signaling module parameters

	Value	Reference	Comment
K1	0.13 µM	[16]	IP3R receptor parameter
K2	30 µM	[18]	IP3R receptor parameter
K3	0.005 µM	[18]	IP3R receptor parameter
K5	0.25 µM	[18]	IP3R receptor parameter
K_{spca}	0.27 µM	[19]	SPCA calcium affinity
k_{leak}	0.1	This work	RNE calcium leak coefficient
k_{IP3R}	1.6 s^{-1}	Estimated from [20]	Single IP3R calcium flux
k_{leakPP}	0.15 µM/s	[14]	Calcium leak into the cytosol
K_{PLC}	0.7 µM	[22]	PLCδ calcium affinity

k_{PLC}	1800 s^{-1}	[22]	PLCδ catalytic constant
N_{PLC}	15	S8	PLCδ copy number
k_{SPCA}	27 s^{-1}	[23]	SPCA turnover constant
N_{SPCA}	370	S8	SPCA copy number
V_{cyt}	18.4 fl	S2	Cytosol volume
N_{IP3R}	1850	S8	IP3R copy number
N_{IP5pp}	240	S8	IP5pp copy number
$k_{catip5pp}$	340 s^{-1}	[25]	IP5pp turnover constant
K_{IP5pp}	0.7 μM	[25]	The affinity of IP5pp for IP_3
N_{CaRet}	54000	S8	Calreticulin copy number
$N_{CaRetSlow}$	$N_{CaRet}\times17$	[26]	Calreticulin Site C capacity
$N_{CaRetFast}$	$N_{CaRet}\times1$	[26]	Calreticulin Site P capacity
$k_{+CaRetSlow}$	0.1 $(\mu M\times s)^{-1}$	[15]	Calreticulin Site C association rate
$K_{dCaRetSlow}$	2000 μM	[15]	Calreticulin Site C Dissociation constant
$k_{+CaRetFast}$	0.05	This work	Calreticulin Site P association rate
$K_{dCaRetFast}$	1 μM	[27]	Calreticulin Site P Dissociation constant
N_{CaM}	18000	S8	Calmodulin copy number
$N_{CaMSlow}$	$N_{CaM}\times2$	[15]	Calmodulin Site N capacity
$N_{CaMFast}$	$N_{CaM}\times2$	[15]	Calmodulin Site C capacity
$k_{+CaMSlow}$	10 s^{-1}	[28]	Calmodulin Site C association rate
$K_{dCaMSlow}$	1 μM	[15]	Calmodulin Site C Dissociation constant
$k_{+CaMFast}$	100 $(\mu M\times s)^{-1}$	[28]	Calmodulin Site N association rate
$k_{-CaMFast}$	500 s^{-1}	[28]	Calmodulin Site N Dissociation constant
V_{pmca}	1 μM/s	[13]	Maximum PMCA calcium extrusion velocity
K_{pmca}	0.2 μM	[29]	PMCA calcium half-activation constant
$CaER_0$	1020 μM	[30]	Initial RNE calcium concentration
$CaIn_0$	0.1 μM	[31]	Initial cytosol calcium concentration
$V_{CatSper}$	0.7 μM/s	This work	Maximum CatSper flux
D_{IP3}	10 $\mu m^2/s$	[32]	IP_3 diffusion coefficient
$P_{CatSper}$	-	From S4	CatSper channel open state probability
β	181	From S2	RNE volume to cytosol volume ratio

S6. Homogeneous model design

The homogenous model (Fig. S5) design and equations are similar to three-dimensional model design, except the diffusion of calcium and IP_3 and restriction of PMCA, CatSper, IP3R, RNEleak, and PLCδ to particular spermatozoa parts are not taken into account. The only two parameters differing from the three-dimensional model are $kcat_{IP3}$ = 100 and k_{leak} = 0.01. The model consists of two compartments, the cytosol, and the RNE. PMCA and CatSper-mediated calcium fluxes, calcium leak, calcium buffering by CaM, IP_3 degradation, and generation are assumed to happen in the cytosol. Calcium buffering by CaRet is assumed to happen in the RNE. RNE_{leak}, SPCA, and IP3R mediate flux between the two compartments.

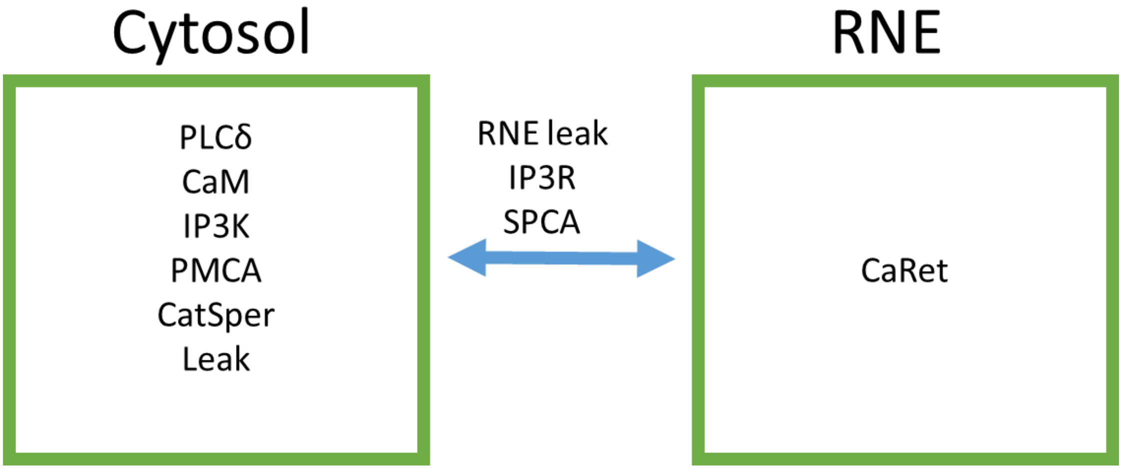

Figure. S5. Homogeneous model design. In the homogeneous model, the diffusion of calcium and IP_3 and restriction of PMCA, CatSper, IP3R, RNEleak, and PLCδ to particular spermatozoa parts are not taken into account. The model consists of two compartments, the cytosol, and the RNE. PMCA and CatSper-mediated calcium fluxes, calcium leak, calcium buffering by CaM, IP_3 degradation, and generation are assumed to happen in the cytosol. Calcium buffering by CaRet is assumed to happen in the RNE. RNEleak, SPCA, and IP3R mediate flux between the two compartments.

S7. Quasi-heterogeneous model

An auxiliary quasi-heterogeneous model in which cytosol was divided into two compartments, cytosol and tail, was built (Fig. S6).

The following equation described calcium flux from PP into cytosol:

$$J_{QuasiDiff} = \alpha([Ca^{2+}_{Head}] - [Ca^{2+}_{PP}]),$$

Where $[Ca^{2+}_{Head}]$ denotes calcium concentration in the sperm head, $[Ca^{2+}_{PP}]$ denotes calcium concentration in sperm PP, and α describes diffusion speed between two compartments.

The model was capable of describing both the single peak and the oscillations. Response type depends on the value of α. For values of α higher than 1, the model described the single peak. For values lower than unity, the model described the oscillations, and their period was dependent on the value of α (Fig. S7). Lower values of α corresponded to lower oscillation frequency. This result is consistent with the results obtained with a three-dimensional model.

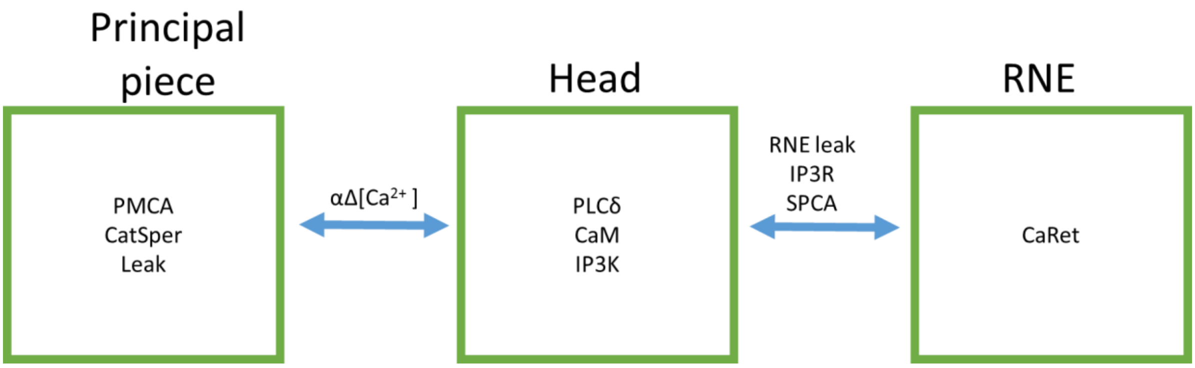

Figure S6. Scheme of the quasi-heterogeneous model. Quasi-heterogenous model design and equations are similar to the homogeneous model, except that in the quasi-heterogeneous model, the cytosol is divided into two compartments – sperm head and sperm principal piece. Calcium influx mediated by CatSper channel, calcium leak from extracellular space, and PMCA-mediated calcium extrusion are assumed to happen in the sperm principal piece. Calcium buffering by CaM, IP_3 generation by PLCδ, and IP_3 metabolism by IP5pp are assumed to happen in the sperm head. Calcium transfer from PP into sperm head is described by mass-action law.

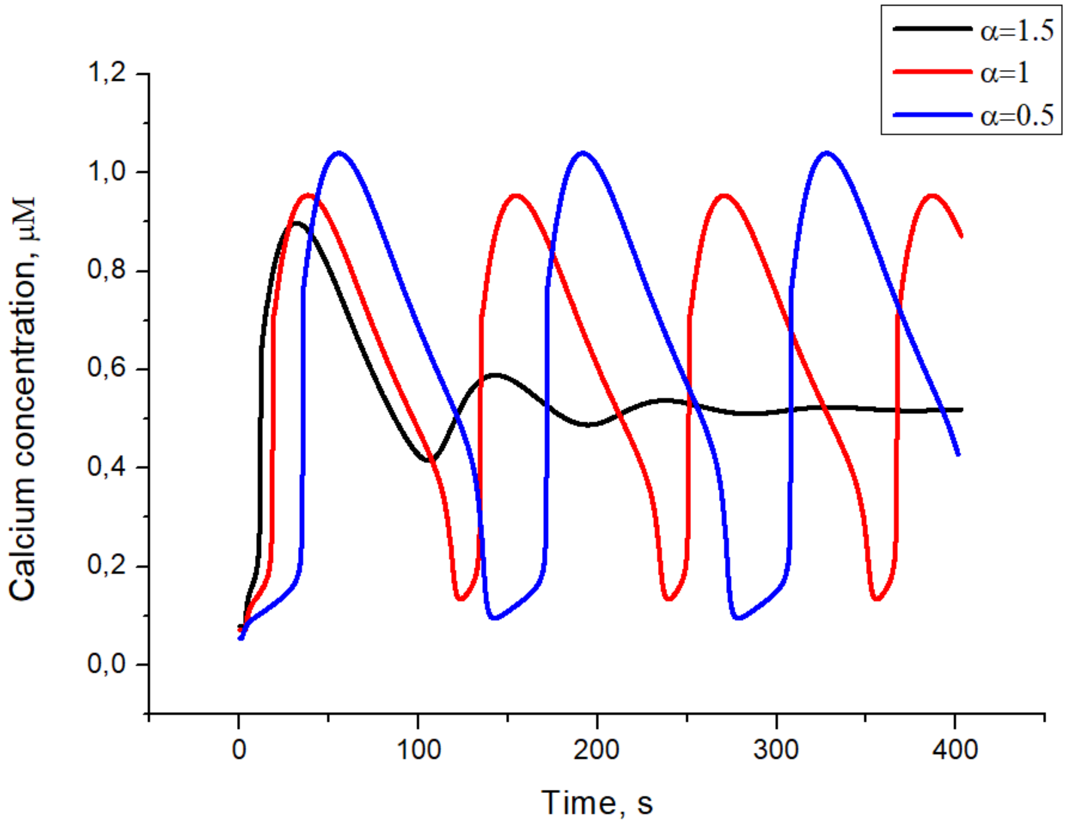

Figure S7. Quasi-heterogeneous model computational experiment on progesterone activation of sperm cells. Progesterone concentration is set equal to 5 μM at t=0. Different colors show computational experiments with different values of parameter α, which governs calcium diffusion between two compartments. Lower values of α describe slower calcium diffusion. For values, lesser than 1, an oscillatory response is observed. For values greater than 1, a single peak is observed.

S8. Protein number estimation

Protein number estimation is conducted in the following fashion: protein abundance in parts per million was taken from pax-db [33] database for either the murine sperm or, if unavailable, human testis dataset. Afterward, the protein abundance was multiplied by the number of proteins in the cell per volume unit (it is assumed to be $10^6/\mu m^3$ [34]) and by spermatozoon volume (18 μm^3 [35]).

S9. Calculating the change in channel flux upon addition of nominally calcium-free buffer

It is known that ion current I through a channel could be calculated from the following equation:

$$I = g(V_m - E),$$

Where g is the conductance of the channel, V_m is the membrane potential and E is the Nernst potential:

$$E = \frac{RT}{zF}\ln\left(\frac{[Ca^{2+}_{Ext}]}{[Ca^{2+}_{Cyt}]}\right).$$

Here z denotes the ion charge and $[Ca^{2+}_{Ext}]$ denotes the extracellular calcium concentration. In [36], $[Ca^{2+}_{Ext}]$ was changed from 2mM to ~5 μM. Membrane potential of capacitated sperm cells is assumed to be -58 mV [37]. Fold-change in all calcium fluxes (CatSper and principal piece leak) was calculated to be 1.7.

Score	Expect	Method	Identities	Positives	Gaps
263 bits(672)	4e-97	Compositional matrix adjust.	123/146(84%)	135/146(92%)	0/146(0%)

```
Query  1    QDMTQPLNHYFICSSHNTYLVGDQLCGQSSVEGYIRALKRGCRCVEVDVWDGPSGEPVVY  60
            QDMTQPL+HY+I SSHNTYLVGDQLCGQSSVEGYIRALKRGCRCVEVD WDGP GEPVVY
Sbjct  1    QDMTQPLSHYYINSSHNTYLVGDQLCGQSSVEGYIRALKRGCRCVEVDTWDGPDGEPVVY  60

Query  61   HGHTLTSRILFKDVVATVAQYAFQTSDYPVILSLETHCSWEQQQTMARHLTEILGEQLLS  120
            HGHTLTSRILFKDV+AT+AQYAFQ+SDYP+ILSLE HC+WEQQ+TMA HLTEILGEQLL
Sbjct  61   HGHTLTSRILFKDVLATLAQYAFQSSDYPLILSLENHCTWEQQRTMAHHLTEILGEQLLR  120

Query  121  TTLDGVLPTQLPSPEELRRKILVKGK  146
             TL+G+L   +PSPE+LR KILVKGK
Sbjct  121  NTLEGLLVDSMPSPEQLRGKILVKGK  146
```

A

Score	Expect	Method	Identities	Positives	Gaps
203 bits(516)	2e-74	Compositional matrix adjust.	95/117(81%)	104/117(88%)	0/117(0%)

```
Query  1    LSSLVIYLKSVSFRSFTHSKEHYHFYEISSFSETKAKRLIKEAGNEFVQHNTWQLSRVYP  60
            LS+LV+YL++V F SFTHSKE+YH Y+ISSFSE+KAK LIKEAGNEFVQHN  QL RVYP
Sbjct  1    LSALVVYLRTVPFCSFTHSKENYHIYDISSFSESKAKNLIKEAGNEFVQHNARQLCRVYP  60
```

B.

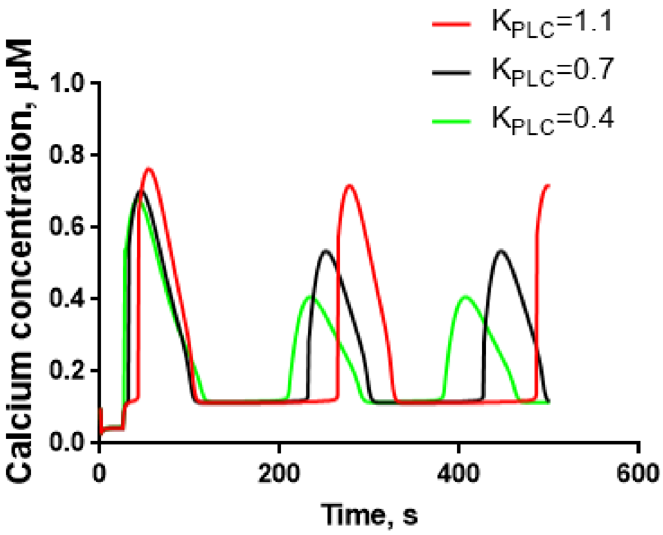

C.

Figure S8. Proof of the validity of PLCd inclusion in the model. The alignment of the aminoacid sequences of murine and human PLCd demonstrates a 88% similarity for Y domain (A) and 92% similarity for X domain (B) (catalytic domains). Thus, the kinetic parameters of these enzymes should not differ much. Variation (C) of the PLCd parameters in the heterogeneous model also does not qualitatively affect the modeling results

S10. Discussion on participation of PLCδ in progesterone activation of human cells

We believe, that there exists strong evidence that PLCδ participates in progesterone activation of human sperm cells. First, it has been shown that PLCδ is present in human sperm cells [38]. Second, it has been shown that calcium ionophore A23187 can induce both acrosome reaction and rapid PIP_2 breakdown [39]. PLC from human sperm extracts is activated by calcium with a half-maximal activity around endogenous calcium concentrations and is inhibited by EGTA [40]. Moreover, response to progesterone is pertussis toxin-insensitive [41] which eliminates the possibility of PLCβ participation. Progesterone-induced calcium oscillations are completely unaltered by U-73122 [36], which is shown to not inhibit PLCδ [42]. The evidence on presence of ryanodine receptors in mammalian spermatozoa is controversial, with some authors stating that they are not present [43]. The following data suggest that PLCδ participates in progesterone-induced calcium activation. Additionally, other isoforms of PLC can also activated by calcium, though to a lesser extent [44].

S11. Parameter scan for the homogeneous model

To determine whether the period of oscillations of hundred seconds or more with the concentration range of 400-1000 nM was possible in the homogeneous model, the sensitivities analysis for model parameters was performed. Parameters which influenced the $[Ca^{2+}_{Cyt}]$the most (K_{spca}, V_{spca}, K_{pmca}, K_{plcd}, V_{plc}, K1, K5) were chosen. For this set of parameters, parameter scan was performed for all the parameters simultaneously. Three different values (3 times lower than the one in the model, the one in the homogeneous model, 3 times higher than the one in the model) were chosen for each parameter. If the resulting model response was oscillatory, oscillations period and maximal concentrations was determined

(Fig. 9). As it could be seen, for the oscillation period to be more than 100s, the maximal oscillations concentration had to be more than 1.5 μM.

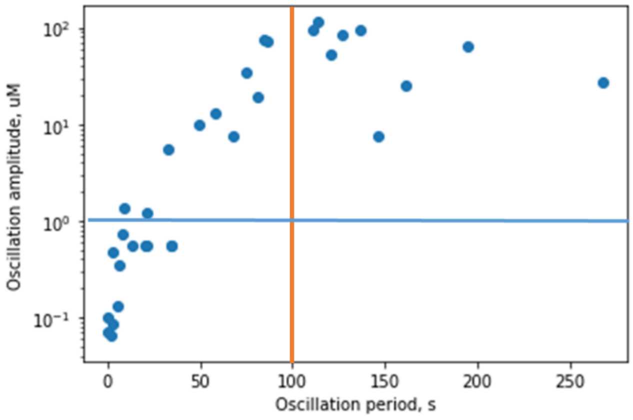

Figure S9. Dependence of calcium oscillation maximal concentration on the oscillation frequency for different model parameter value. Orange line denotes the oscillation period of 100 s. Blue line denotes the oscillation maximal concentration of 1000 nM.

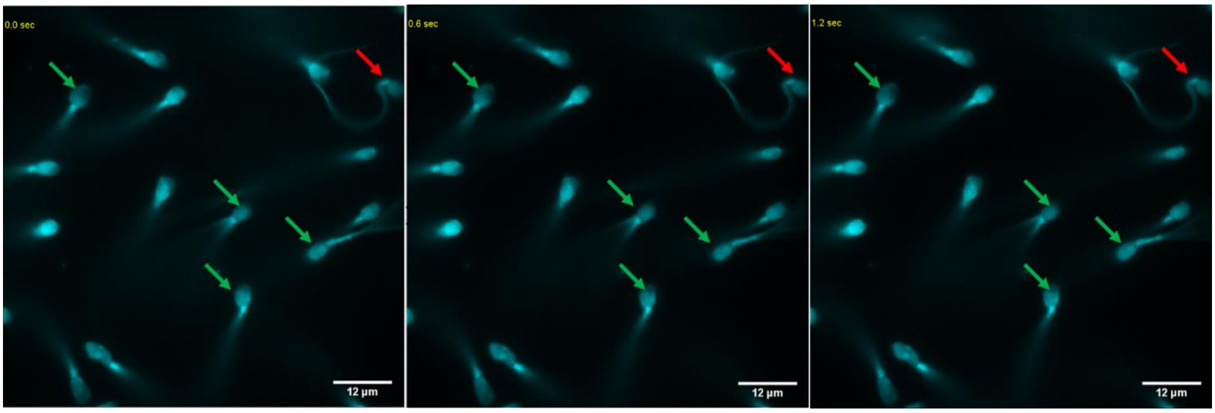

Figure S10. Vitality check for the spermatozoa adherent to poly-L-lysine. Sperm viability was checked through looking at the Fura-RED-loaded sperm tail movement. If it was possible to see a rapidly moving sperm flagellum forming a "triangle" shade, the sperm cell was assumed to be viable. Green arrows denote the viable sperm cells. Red arrow denotes the immotile sperm cell. LUTs were adjusted automatically using ImageJ. The images correspond to calcium-free Fura-RED excited with 488 nm laser.

S12. Adhesion of different populations of sperm cells to poly-L-lysine

Poly-L-lysine is widely used as an adhesion molecule for sperm cells in calcium signaling studies [45]–[48]. It is not clear yet whether the functional state of the sperm cell may influence the adhesion to Poly-L-lysine. For example, only the spermatozoa with the highest fertilization rates attach to bovine fallopian tubes epithelium [49]. However, we can conclude that if just a subpopulation of cells is capable of attaching to poly-L-lysine, it is the major one: binding to Poly-L-Lysine coated glass is 66% effective compared to agarose [50] (which immobilizes all the cells adjacent to the glass regardless of their functional state), and does not differ significantly from binding to laminin-coated glass [50]. Even if only a subpopulation of cells is capable of binding to poly-L-lysine, we are examining a major subpopulation of sperm cells.

S13. Supporting references

[1] F. A. Balabin, D. S. Morozova, A. S. Mayorov, A. A. Martyanov, M. A. Panteleev, and A. N. Sveshnikova, "Clusterization of Inositol Trisphosphate Receptors Determines the Shape of the Calcium Oscillation Peak in Platelet Cytosol," *Moscow Univ. Phys. Bull.*, vol. 73, no. 5, pp. 526–533, Sep. 2018, doi: 10.3103/S0027134918050041.

[2] C. Serres, D. Escalier, and G. David, "Ultrastructural morphometry of the human sperm flagellum with a stereological analysis of the lengths of the dense fibres," *Biol. Cell*, vol. 49, no. 2, pp. 153–161, 1984, doi: 10.1111/j.1768-322X.1984.tb00233.x.

[3] J. A. Mossman, J. T. Pearson, H. D. Moore, and A. A. Pacey, "Variation in mean human sperm length is linked with semen characteristics," *Hum. Reprod.*, vol. 28, no. 1, pp. 22–32, Jan. 2013, doi: 10.1093/humrep/des382.

[4] P. Sunanda, B. Panda, C. Dash, R. N. Padhy, and P. Routray, "An illustration of human sperm morphology and their functional ability among different group of subfertile males," *Andrology*, vol. 6, no. 5, pp. 680–689, Sep. 2018, doi: 10.1111/andr.12500.

[5] H.-C. Ho and S. S. Suarez, "Characterization of the Intracellular Calcium Store at the Base of the Sperm Flagellum That Regulates Hyperactivated Motility1," *Biol. Reprod.*, vol. 68, no. 5, pp. 1590–1596, May 2003, doi: 10.1095/biolreprod.102.011320.

[6] N. Laufer, S. Segal, H. Yaffe, H. Svartz, and N. B. Grover, "Volume And Shape Of Normal Human Spermatozoa*," *Fertil. Steril.*, vol. 28, no. 4, pp. 456–458, Apr. 1977, doi: 10.1016/S0015-0282(16)42497-0.

[7] M. R. Miller *et al.*, "Unconventional endocannabinoid signaling governs sperm activation via the sex hormone progesterone," *Science (80-.).*, vol. 352, no. 6285, pp. 555–559, Apr. 2016, doi: 10.1126/science.aad6887.

[8] S. Tyukhtenko *et al.*, "Specific Inter-residue Interactions as Determinants of Human Monoacylglycerol Lipase Catalytic Competency," *J. Biol. Chem.*, vol. 291, no. 6, pp. 2556–2565, Feb. 2016, doi: 10.1074/jbc.M115.670257.

[9] J. D. Knight, M. G. Lerner, J. G. Marcano-Velázquez, R. W. Pastor, and J. J. Falke, "Single Molecule Diffusion of Membrane-Bound Proteins: Window into Lipid Contacts and Bilayer Dynamics," *Biophys. J.*, vol. 99, no. 9, pp. 2879–2887, Nov. 2010, doi: 10.1016/j.bpj.2010.08.046.

[10] C. A. Wolfe, P. S. James, A. R. Mackie, S. Ladha, and R. Jones, "Regionalized Lipid Diffusion in the Plasma Membrane of Mammalian Spermatozoa," *Biol. Reprod.*, vol. 59, no. 6, pp. 1506–1514, Dec. 1998, doi: 10.1095/biolreprod59.6.1506.

[11] P. V Lishko, I. L. Botchkina, and Y. Kirichok, "Progesterone activates the principal Ca2+ channel of human sperm.," *Nature*, vol. 471, no. 7338, pp. 387–91, Mar. 2011, doi: 10.1038/nature09767.

[12] J. Correia, F. Michelangeli, and S. Publicover, "Regulation and roles of Ca2+ stores in human sperm," *REPRODUCTION*, vol. 150, no. 2, pp. R65–R76, Aug. 2015, doi: 10.1530/REP-15-0102.

[13] G. Wennemuth, D. F. Babcock, and B. Hille, "Calcium Clearance Mechanisms of Mouse Sperm," *J. Gen. Physiol.*, vol. 122, no. 1, pp. 115–128, Jul. 2003, doi: 10.1085/jgp.200308839.

[14] L.-F. Li, C. Xiang, Y.-B. Zhu, and K.-R. Qin, "Modeling of progesterone-induced intracellular calcium signaling in human spermatozoa," *J. Theor. Biol.*, vol. 351, pp. 58–66, Jun. 2014, doi: 10.1016/j.jtbi.2014.02.026.

[15] S. Means *et al.*, "Reaction Diffusion Modeling of Calcium Dynamics with Realistic ER Geometry," *Biophys. J.*, vol. 91, no. 2, pp. 537–557, Jul. 2006, doi: 10.1529/biophysj.105.075036.

[16] G. W. De Young and J. Keizer, "A single-pool inositol 1,4,5-trisphosphate-receptor-based model for agonist-stimulated oscillations in Ca2+ concentration.," *Proc. Natl. Acad. Sci.*, vol. 89, no. 20, pp. 9895–9899, Oct. 1992, doi: 10.1073/pnas.89.20.9895.

[17] T. Kawai *et al.*, "Polarized PtdIns(4,5)P 2 distribution mediated by a voltage-sensing phosphatase (VSP) regulates sperm motility," *Proc. Natl. Acad. Sci.*, vol. 116, no. 51, pp. 26020–26028, Dec. 2019, doi: 10.1073/pnas.1916867116.

[18] X. Cai, X. Li, H. Qi, F. Wei, J. Chen, and J. Shuai, "Comparison of gating dynamics of different IP 3 R channels with immune algorithm searching for channel parameter distributions," *Phys. Biol.*, vol. 13, no. 5, p. 056005, Oct. 2016, doi: 10.1088/1478-3975/13/5/056005.

[19] P. C. Chandrasekera, M. E. Kargacin, J. P. Deans, and J. Lytton, "Determination of apparent calcium affinity for endogenously expressed human sarco(endo)plasmic reticulum calcium-ATPase isoform SERCA3," *Am. J. Physiol. Cell Physiol.*, vol. 296, no. 5, pp. C1105-1114, May 2009, doi: 10.1152/ajpcell.00650.2008.

[20] H. Vais, J. K. Foskett, and D.-O. Daniel Mak, "Unitary Ca2+ current through recombinant type 3 InsP3 receptor channels under physiological ionic conditions," *J. Gen. Physiol.*, vol. 136, no. 6, pp. 687–700, Dec. 2010, doi: 10.1085/jgp.201010513.

[21] A. Atri, J. Amundson, D. Clapham, and J. Sneyd, "A single-pool model for intracellular calcium oscillations and waves in the Xenopus laevis oocyte," *Biophys. J.*, vol. 65, no. 4, pp. 1727–1739, Oct. 1993, doi: 10.1016/S0006-3495(93)81191-3.

[22] Y. Banno and Y. Nozawa, "Characterization of partially purified phospholipase C from human platelet membranes," *Biochem. J.*, vol. 248, no. 1, pp. 95–101, Nov. 1987, doi: 10.1042/bj2480095.

[23] L.-H. Li, X.-R. Tian, Z. Jiang, L.-W. Zeng, W.-F. He, and Z.-P. Hu, "The Golgi Apparatus: Panel Point of Cytosolic Ca 2+ Regulation," *Neurosignals*, vol. 21, no. 3–4, pp. 272–284, 2013, doi: 10.1159/000350471.

[24] B. VERJANS, R. LECOCQ, C. MOREAU, and C. ERNEUX, "Purification of bovine brain inositol-1,4,5-trisphosphate 5-phosphatase," *Eur. J. Biochem.*, vol. 204, no. 3, pp. 1083–1087, Mar. 1992, doi: 10.1111/j.1432-1033.1992.tb16732.x.

[25] M. De Pittà, M. Goldberg, V. Volman, H. Berry, and E. Ben-Jacob, "Glutamate regulation of calcium and IP3 oscillating and pulsating dynamics in astrocytes," *J. Biol. Phys.*, vol. 35, no. 4, pp. 383–411, Oct. 2009, doi: 10.1007/s10867-009-9155-y.

[26] S. Baksh and M. Michalak, "Expression of calreticulin in Escherichia coli and identification of its Ca2+ binding domains.," *J. Biol. Chem.*, vol. 266, no. 32, pp. 21458–65, Nov. 1991.

[27] M. Michalak, E. F. Corbett, N. Mesaeli, K. Nakamura, and M. Opas, "Calreticulin: one protein, one gene, many functions.," *Biochem. J.*, vol. 344 Pt 2, pp. 281–92, Dec. 1999.

[28] L. Hoffman, A. Chandrasekar, X. Wang, J. A. Putkey, and M. N. Waxham, "Neurogranin Alters the Structure and Calcium Binding Properties of Calmodulin," *J. Biol. Chem.*, vol. 289, no. 21, pp. 14644–14655, May 2014, doi: 10.1074/jbc.M114.560656.

[29] A. R. Penheiter, A. J. Caride, Á. Enyedi, and J. T. Penniston, "Tryptophan 1093 Is Largely

Responsible for the Slow Off Rate of Calmodulin from Plasma Membrane Ca2+ Pump 4b," *J. Biol. Chem.*, vol. 277, no. 20, pp. 17728–17732, May 2002, doi: 10.1074/jbc.M111608200.

[30] S. B. Herrick, D. L. Schweissinger, S.-W. Kim, K. R. Bayan, S. Mann, and R. A. Cardullo, "The acrosomal vesicle of mouse sperm is a calcium store," *J. Cell. Physiol.*, vol. 202, no. 3, pp. 663–671, Mar. 2005, doi: 10.1002/jcp.20172.

[31] S. D. Olson, S. S. Suarez, and L. J. Fauci, "A Model of CatSper Channel Mediated Calcium Dynamics in Mammalian Spermatozoa," *Bull. Math. Biol.*, vol. 72, no. 8, pp. 1925–1946, Nov. 2010, doi: 10.1007/s11538-010-9516-5.

[32] G. D. Dickinson, K. L. Ellefsen, S. P. Dawson, J. E. Pearson, and I. Parker, "Hindered cytoplasmic diffusion of inositol trisphosphate restricts its cellular range of action," *Sci. Signal.*, vol. 9, no. 453, pp. ra108–ra108, Nov. 2016, doi: 10.1126/scisignal.aag1625.

[33] M. Wang *et al.*, "PaxDb, a Database of Protein Abundance Averages Across All Three Domains of Life," *Mol. Cell. Proteomics*, vol. 11, no. 8, pp. 492–500, Aug. 2012, doi: 10.1074/mcp.O111.014704.

[34] R. Milo, "What is the total number of protein molecules per cell volume? A call to rethink some published values," *BioEssays*, vol. 35, no. 12, pp. 1050–1055, Dec. 2013, doi: 10.1002/bies.201300066.

[35] N. Laufer, S. Segal, H. Yaffe, H. Svartz, and N. B. Grover, "Volume And Shape Of Normal Human Spermatozoa*," *Fertil. Steril.*, vol. 28, no. 4, pp. 456–458, Apr. 1977, doi: 10.1016/S0015-0282(16)42497-0.

[36] C. V. Harper, C. L. R. Barratt, and S. J. Publicover, "Stimulation of Human Spermatozoa with Progesterone Gradients to Simulate Approach to the Oocyte," *J. Biol. Chem.*, vol. 279, no. 44, pp. 46315–46325, Oct. 2004, doi: 10.1074/jbc.M401194200.

[37] I. López-González *et al.*, "Membrane hyperpolarization during human sperm capacitation," *MHR Basic Sci. Reprod. Med.*, vol. 20, no. 7, pp. 619–629, Jul. 2014, doi: 10.1093/molehr/gau029.

[38] I. Urizar-Arenaza *et al.*, "Phosphoproteomic and Functional Analyses Reveal Sperm-specific Protein Changes Downstream of Kappa Opioid Receptor in Human Spermatozoa," *Mol. Cell. Proteomics*, vol. 18, no. Supplement 1, pp. S118–S131, Mar. 2019, doi: 10.1074/mcp.RA118.001133.

[39] E. R. S. Roldan and R. A. P. Harrison, "Polyphosphoinositide breakdown and subsequent exocytosis in the Ca2+/ionophore-induced acrosome reaction of mammalian spermatozoa," *Biochem. J.*, vol. 259, no. 2, pp. 397–406, Apr. 1989, doi: 10.1042/bj2590397.

[40] H. Ribbes, M. Plantavid, P. J. Bennet, H. Chap, and L. Douste-Blazy, "Phospholipase C from human sperm specific for phosphoinositides," *Biochim. Biophys. Acta - Lipids Lipid Metab.*, vol. 919, no. 3, pp. 245–254, Jun. 1987, doi: 10.1016/0005-2760(87)90264-5.

[41] J. Tesarik, A. Carreras, and C. Mendoza, "Differential sensitivity of progesterone- and zona pellucida-induced acrosome reactions to pertussis toxin," *Mol. Reprod. Dev.*, vol. 34, no. 2, pp. 183–189, Feb. 1993, doi: 10.1002/mrd.1080340210.

[42] R. R. Klein *et al.*, "Direct Activation of Human Phospholipase C by Its Well Known Inhibitor U73122," *J. Biol. Chem.*, vol. 286, no. 14, pp. 12407–12416, Apr. 2011, doi: 10.1074/jbc.M110.191783.

[43] H.-C. Ho and S. S. Suarez, "An Inositol 1,4,5-Trisphosphate Receptor-Gated Intracellular Ca2+ Store Is Involved in Regulating Sperm Hyperactivated Motility1," *Biol. Reprod.*, vol. 65, no. 5, pp. 1606–1615, Nov. 2001, doi: 10.1095/biolreprod65.5.1606.

[44] V. ALLEN, P. SWIGART, R. CHEUNG, S. COCKCROFT, and M. KATAN, "Regulation of inositol lipid-specific phospholipase Cδ by changes in Ca2+ ion concentrations," *Biochem. J.*, vol. 327, no. 2, pp. 545–552, Oct. 1997, doi: 10.1042/bj3270545.
